# Cognitive, Functional, and Emotional Recovery in Patients with Stroke: A Multidimensional Prospective Analysis

**DOI:** 10.3390/neurolint17100164

**Published:** 2025-10-08

**Authors:** Emilio Rubén Pego Pérez, Lourdes Bermello López, Eva Gómez Fernández, María del Rosario Marín Arnés, Mercedes Fernández Vázquez, Carlota Touza González, María Irene Núñez Hernández

**Affiliations:** 1 Departament of Psychiatry, Radiology, Public Health, Nursing and Medicine, Faculty of Nursing, University of Santiago de Compostela, 15704 Santiago de Compostela, Spain; 2 Research Group of Dependencie, Gerontology y Geriatrics, Faculty of Nursing, University of Santiago de Compostela, 15704 Santiago de Compostela, Spain; 3 Neurology and Neurosurgery of Lucus Augusti Hospital, 27003 Lugo, Spain; lubermello@hotmail.com (L.B.L.); eva.gomez.fernandez@sergas.es (E.G.F.); charochili@yahoo.es (M.d.R.M.A.); mercedes.fernandez.vazquez@sergas.es (M.F.V.); irene.nunez.hernandez@sergas.es (M.I.N.H.); 4 Galician Health Service, Santiago de Compostela Hospital, 15704 Santiago de Compostela, Spain; carlota.touza.gonzalez@sergas.es

**Keywords:** ischemic stroke, transient ischemic attack, tissue plasminogen activator, functional status, cognitive dysfunction, MMSE, mini mental state examination, depression, surveys and questionnaires

## Abstract

Background: Stroke is a major cerebrovascular disease characterized by disrupted cerebral blood flow, leading to neuronal damage and significant physical, cognitive, and emotional sequelae. While advancements in acute stroke management have improved survival rates, long-term complications such as cognitive impairment and depression continue to hinder recovery. This study addresses these dimensions within the context of ischemic stroke. Aim: The aim of this study was to analyze the cognitive status, functionality, and depressive symptoms in patients with ischemic stroke, exploring interrelations between cognitive, functional, and emotional outcomes to prioritize clinical interventions. Design: This was an analytical, observational, cohort, and prospective study. Methods: The study included 81 subjects diagnosed with ischemic stroke admitted to the Neurology Department of Lucus Augusti University Hospital. Data were collected at three time points—admission, discharge, and follow-up—using validated instruments such as the National Institutes of Health Stroke Scale, Mini-Mental State Examination, Barthel Index, and Beck Depression Inventory. Statistical analyses included Spearman’s correlation, Kruskal–Wallis, and Mann–Whitney tests. Results: Patients with greater cognitive impairment at admission showed poorer functional recovery and higher depressive symptoms during follow-up. Depressive symptoms remained minimal in most cases, but correlations with cognitive and functional deficits were significant. NIHSS scores at admission strongly predicted both functional and emotional recovery, reinforcing its value in early prognosis and therapeutic planning. Conclusions: This study highlights the importance of integrating cognitive, functional, and emotional dimensions into stroke care protocols to optimize patient recovery and improve long-term outcomes.

## 1. Background

Stroke involves a transient or permanent interruption of cerebral blood flow, depriving neurons of oxygen and glucose essential for their function. This leads to neuronal death and significant physical, cognitive, and emotional sequelae. Based on the mechanism of vascular disruption, stroke is classified into two main types: ischemic (approximately 80% of cases) and hemorrhagic [1,2].

Ischemic stroke results from the obstruction of a cerebral artery, commonly due to thrombi or emboli formation. This type of stroke may present as a transient ischemic attack (TIA), lasting less than 24 h, or as a cerebral infarction, which exceeds this duration. The primary symptoms include localized muscle weakness, speech difficulties, sensory loss, and visual disturbances, which may manifest as diplopia or sudden, severe headaches. These symptoms, along with the severity of the sequelae, make stroke one of the leading causes of disability and mortality worldwide [1,2,3].

Acute ischemic stroke is a heterogeneous disease, and differentiating its subtypes (cardioembolic stroke, lacunar infarct, infarct of unusual etiology, essential cerebral infarct, atherothrombotic infarct) in clinical studies is crucial. Stroke subtypes significantly impact the distribution of risk factors, stroke severity, and outcomes, making their adequate differentiation essential for improving clinical management and research protocols [4].

Stroke ranks among the leading causes of global disability, with annual figures surpassing those of other major neurological conditions. According to the World Health Organization (WHO), more than 12 million new cases of stroke occur worldwide each year. It ranks as the third leading cause of mortality, the primary cause of disability, and the second leading cause of dementia among adults in developed countries [5,6,7].

In Spain, stroke causes approximately 40,000 deaths annually, and it is estimated that a new case occurs every six minutes. Furthermore, two out of every three survivors experience some form of disabling sequela, and only 40% achieve full autonomy recovery. These statistics emphasize the urgency of developing robust prevention, acute care, and recovery protocols to mitigate stroke-related morbidity and mortality [1,3,7].

Stroke incidence increases with age, doubling every decade after 55 years and tripling in individuals over 80 years old. While men show a higher prevalence of ischemic stroke, women experience more disabling sequelae and higher mortality rates, highlighting sex-specific healthcare needs. Major risk factors include metabolic conditions (diabetes mellitus, obesity, dyslipidemia), arterial hypertension (present in 70% of cases), sedentary lifestyle, and substance use such as tobacco and alcohol [1,2,3].

Stroke poses a substantial economic burden, accounting for 2% to 4% of total healthcare expenditure in industrialized countries, a figure comparable to other major chronic conditions such as cardiovascular disease. In Spain, direct treatment costs during the first three months reach approximately €4000 per person, while indirect costs, driven by long-term disability and cognitive impairments, have a greater economic impact, reducing quality of life and productivity [3,6].

Intravenous thrombolysis with tissue plasminogen activator (rt-PA) and mechanical thrombectomy are two key interventions for ischemic stroke treatment, both effective in arterial recanalization and reducing long-term sequelae. The efficacy of rt-PA is highly time-sensitive, with optimal results achieved within the first 4.5 h after symptom onset, highlighting the need for rapid diagnosis and intervention. Mechanical thrombectomy has emerged as a critical option for patients ineligible for thrombolysis or those unresponsive to thrombolytic therapy, significantly improving recanalization rates and outcomes. Together, these interventions have transformed the landscape of acute ischemic stroke care, offering improved survival and reduced disability [6,7,8,9,10].

Despite advances in acute stroke management, post-stroke complications such as cognitive impairment and depression remain significant challenges due to their complex etiology and variability in recovery trajectories. Vascular cognitive impairment affects up to 60% of patients with stroke within the first year after stroke, with one-third progressing to long-term dementia, underscoring its clinical significance. These complications not only elevate healthcare costs but also increase caregiver burden, particularly in regions with limited access to rehabilitation services. Post-stroke depression, affecting approximately one-third of survivors, is associated with poorer rehabilitation adherence, reduced quality of life, heightened recurrence risk, and increased mortality rates. Addressing cognitive, emotional, and functional impairments is vital for achieving holistic post-stroke recovery and improving patient outcomes [11,12,13,14,15].

Historically, stroke management has focused on sensory–motor sequelae, often neglecting neuropsychiatric complications such as cognitive impairment and depression. Cognitive impairment occurs in up to 30% of patients during the acute phase, with prevalence varying across cohorts and stroke subtypes. Depression significantly impairs functional recovery by reducing patient motivation and adherence to rehabilitation programs. Both cognitive impairment and depression profoundly affect autonomy, rehabilitation motivation, and quality of life, reinforcing the need for a multidimensional approach to post-stroke care. These impairments delay functional recovery and diminish the likelihood of achieving full independence, particularly among older people with limited resilience [11,12].

To address these needs, the Lugo, A Mariña, and Monforte de Lemos Health Area, in collaboration with the Investén group from the Carlos III Health Institute (ISCIII), implemented the Registered Nurses’ Association of Ontario (RNAO) best practice guideline for stroke management in 2018. This guideline includes evidence-based recommendations for the functional, cognitive, and emotional assessment of post-stroke patients. Since 2024, targeted strategies for screening cognitive status and depressive symptoms have been introduced at the Lucus Augusti University Hospital (HULA), aiming to enhance care and rehabilitation protocols [16].

The main objective is to analyze cognitive status, functionality, and depressive symptoms in patients with ischemic stroke treated at HULA. The specific objectives are (1) to determine the level of cognitive status, functional dependency, and depressive symptoms, (2) to correlate cognitive status with depressive symptoms and functionality, (3) to relate depressive symptoms to cognitive status and functionality, and (4) to correlate functionality with cognitive status.

## 2. Methods

### 2.1. Design

This was an analytical, observational, exposure-based, cohort, and prospective study.

### 2.2. Population

For the development of this study, the target population selected consists of patients with a clinical diagnosis of ischemic stroke in the Lugo, A Mariña, and Monforte de Lemos health area.

#### Sample and Inclusion/Exclusion Criteria

This study included patients diagnosed with ischemic stroke treated in the Neurology ward at HULA. Inclusion criteria comprised anterior or posterior cerebral occlusion and age ≥ 18 years, while exclusion criteria included intracranial hemorrhage, pregnancy, and cognitive dysfunction that impeded participation.

The Stroke Atlas of Galicia (2018) [17] reported a regional population of 2,701,819 inhabitants. Based on the incidence rate from the IBERICTUS study (187.4 cases per 100,000 inhabitants), approximately 5064 new stroke cases occur annually in Galicia. In Lugo province, with 327,946 inhabitants, this incidence corresponds to roughly 614 cases per year, of which 80% (≈492) are ischemic strokes.

A minimum sample size of 81 subjects was calculated to ensure representativeness of the stroke population in Lugo province. This estimation, based on a 95% confidence interval and a 10% margin of error, guarantees sufficient statistical precision for analyzing cognitive, functional, and emotional outcomes [17].

### 2.3. Variables

#### 2.3.1. Independent Variables

Sociodemographic variables: Age (<65 years, 65–80 years, >80 years), Sex (male, female), Date of ischemic event (mm/yyyy).

Clinical variables: Treatment modality (received rt-PA, received mechanical thrombectomy, or did not receive antithrombotic treatment), Number of falls, Pain level, Presence of pressure ulcers (PU), Presence of aspiration pneumonia, Vital signs (temperature, blood pressure, and blood glucose levels).

#### 2.3.2. Dependent Variables

Dependent variables were neurological and Cognitive status, Depression symptomatology and functional independence.

### 2.4. Instruments

#### 2.4.1. National Institutes Health Stroke Scale (NIHSS)

The NIHSS, developed in 1989, is a widely used tool for assessing stroke severity and its neurological effects, both during the initial evaluation of the patient and throughout their follow-up. It consists of 11 items that evaluate aspects such as level of consciousness, language, vision, and sensory-motor impairment, assigning scores based on the degree of dysfunction.

The total score was calculated by summing the individual scores of each item, with a range from 0 (normal function) to 42 (maximum impairment). Based on the scale’s categorization, patients were classified into asymptomatic (0 points), minimal deficit (1 point), mild deficit (2–5 points), moderate deficit (6–15 points), significant deficit (16–20 points), and severe deficit (>20 points) categories [6].

#### 2.4.2. Beck Depression Inventory (BDI)

The BDI, originally published by Beck et al. in 1996 and later adapted for use in Spain, is a tool designed to assess the severity of depressive symptoms. It consists of 21 items that explore symptoms experienced in the past few weeks, assigning scores from 0 to 3, where 0 indicates lower severity and 3 indicates higher severity.

The total score ranges from 0 to 63, with 14 points as the threshold for considering clinically significant depression. The application of this scale requires approximately 10 min. Based on the total score, symptoms are classified into four categories: no depression (0–13 points), mild depression (14–19 points), moderate depression (20–28 points), and severe depression (29–63 points) [18,19].

#### 2.4.3. Mini-Mental State Examination

The Mini-Mental State Examination, developed by Marshal Folstein in 1975 and adapted to the Spanish version in 1979 by Lobo as the Mini-Cognitive Examination (MEC), is a tool used to assess cognitive impairment, facilitating the detection of disorders such as dementia. The test covers various areas: temporal and spatial orientation, immediate memory, calculation and concentration, delayed memory, language (naming, repetition, comprehension, reading, and writing), and graphic constructive praxis.

The maximum score is 30 points, indicating the absence of cognitive impairment, while a score below 23 points suggests cognitive deterioration. The test takes approximately 5 min to administrate. The scoring categories are: No cognitive impairment (30–27 points); Doubtful cognitive status (26–25 points); Mild-to-moderate cognitive impairment (24–10 points); Moderate-to-severe cognitive impairment (9–6 points), and Severe cognitive impairment (<6 points) [20].

#### 2.4.4. Barthel Index

The Barthel Index, also referred to as the Maryland Disability Index, was developed in 1965 by Mahoney and Barthel and is one of the most widely used tools for assessing the functional capacity of patients. This scale evaluates independence in basic activities of daily living (BADLs), categorizing the level of dependency based on the patient’s degree of autonomy. The instrument assesses 10 BADLs, divided into two categories: seven related to personal care (eating, bathing, dressing, grooming, bowel control, bladder control, and toilet use) and three associated with mobility (walking, transferring, and climbing/descending stairs).

Each activity is scored according to the level of independence, with values ranging from 0 (total dependence) to 15 (total independence). The total score ranges from 0 to 100, where 100 points indicate complete independence and 0 absolute dependence. Based on the score obtained, the level of dependency is classified as follows: 100 points for independence, ≤60 for mild dependence, 40–55 for moderate dependence, 20–35 for severe dependence, and 5–20 for total dependence [21,22].

### 2.5. Data Management

The data collected for this research were completed by the principal investigator and were only accessible to them and the project’s collaborative team. No external individuals had the ability to modify the data. The data were gathered at three specific time points: upon subject admission (neurological evaluation using the NIHSS), at discharge from the Neurology Unit, and during the follow-up consultation at three months.

For the evaluations at discharge and three months, the MEC was used to assess cognitive status, the Barthel Index to evaluate functionality, and the BDI to assess depressive symptoms. These scales were administered by trained nurses, and the data were recorded in a collection notebook specifically designed for this study. The information was stored anonymously and reviewed to ensure its quality and consistency.

### 2.6. Confidentiality of Data and Ethical Considerations

Initiated in 2018 by HULA in collaboration with the Investén Group of the Instituto de Salud Carlos III, this study received ethical approval from the Santiago-Lugo Ethics Committee (Registration Code: 2024-472). The study strictly adhered to the ethical principles outlined in the Declaration of Helsinki and complied with RD 1090/2015 regulations governing clinical trials in Spain, ensuring the protection of participants’ rights and well-being.

Strict measures of anonymization and dissociation were implemented to ensure compliance with relevant data protection laws, including the Spanish Organic Law 3/2018 and associated regulations. Clinical data were recorded in coded case report forms (CRFs), accessible only to the research team and health authorities under confidentiality obligations. Data shared externally were anonymized, and informed consent was obtained from all participants.

The database was fully anonymized, and data will be destroyed or retained in an anonymized form upon study completion, as stipulated in the consent form. Data processing remains under HULA’s responsibility.

### 2.7. Data Analysis

A descriptive analysis was performed for quantitative variables using measures of central tendency, such as the mean (M), and dispersion, such as the standard deviation (SD). For qualitative variables, absolute frequencies and percentages were calculated. The Chi-square test was applied to assess relationships between qualitative variables.

The normality of quantitative variables was evaluated using the Kolmogorov–Smirnov test, revealing a non-parametric distribution for all variables. Consequently, the Kruskal–Wallis test was employed for comparisons across more than two groups, while pairwise differences were analyzed using the Mann–Whitney U test. The strength and direction of associations were assessed using Spearman’s correlation coefficient.

All statistical analyses were conducted using PASW Statistics software (version 23.0; SPSS Inc., Chicago, Illinois, USA), with a bilateral significance level set at *p* < 0.05.

## 3. Results

### 3.1. Sociodemographic and Clinical

The study included 81 subjects diagnosed with ischemic stroke, treated at the HULA. The mean age was 72.43 ± 11.72 years, ranging from 41 to 95 years (Table 1).

#### 3.1.1. Sex, Age and Stay in Hospital

The only variable showing a statistically significant difference between men and women was the risk of depression at discharge (U = 815.00; Z = −2.140; *p* = 0.032), suggesting a distinct emotional impact between sexes. No significant differences were observed for other variables.

Significant differences were identified in functional recovery, as measured by the Barthel Index, both at discharge (χ^2^ = 11.457, df = 2, *p* = 0.003) and at follow-up (χ^2^ = 11.304, df = 2, *p* = 0.004). Younger people (< 65 years) achieved the highest mean ranks, followed by adults (65–80 years) and older people (>80 years), indicating better functional recovery in younger individuals.

Similarly, significant differences were observed in cognitive performance, as measured by MEC scores at discharge (χ^2^ = 15.570, df = 2, *p* < 0.001) and at follow-up (χ^2^ = 19.931, df = 2, *p* < 0.001). Younger people had higher mean ranks compared to adults and older people, suggesting progressive cognitive decline associated with aging.

No relationships were found between hospital length of stay and the dependent variables analyzed.

#### 3.1.2. Age and Ischemic Stroke Group

A weak negative correlation was observed between age categories and Mini-Mental at discharge (r = −0.244; *p* = 0.028). Also, analyses revealed significant differences (χ^2^ = 10.209; df = 4; *p* = 0.037), suggesting that younger people were more likely to achieve higher Mini-Mental scores at discharge.

A moderate negative correlation was observed between age categories and Mini-Mental at follow-up (r = −0.320; *p* = 0.004). Significant differences were analyzed (χ^2^ = 12.827; df = 4; *p* = 0.012). Younger subjects were more likely to achieve higher Mini-Mental scores, indicating better cognitive function at follow-up.

A moderate negative correlation was observed between age categories and functional independence at discharge (r = −0.351; *p* = 0.001). Differences between groups were found (χ^2^ = 12.487; df = 4; *p* = 0.014); younger people were more likely to achieve higher Barthel scores, reflecting greater functional independence at discharge.

A moderate negative correlation was identified between age categories and functional independence at follow-up (r = −0.334; *p* = 0.002). Differences between groups (χ^2^ = 11.650; df = 4; *p* = 0.020) showed that the younger group consistently demonstrated higher levels of functional independence at follow-up, suggesting age as a relevant factor in initial functional status.

#### 3.1.3. Stroke Type and Subtype

Significant differences were observed in initial clinical severity, measured by the NIHSS, between TIA and ischemic stroke groups (U = 153.500, Z = −5.322, *p* < 0.001). Patients with ischemic stroke had higher mean ranks (57.10 vs. 18.03), reflecting greater initial clinical severity in this group. No significant differences were found for other variables.

No significant differences were found between ischemic stroke subtypes and the dependent variables analyzed.

#### 3.1.4. Stroke Subtype in Stroke Group

A statistically significant association was observed between ischemic stroke subtypes and Mini-Mental scores during the follow-up (*p* = 0.031). Specifically, the highest mean rank (43.29) was observed in subtype “Essential cerebral infarct”, while subtype “Cardioembolic stroke” showed the lowest mean rank (29.47), indicating worse cognitive performance in this group. Subtypes “Lacunar infarct” and “Atherothrombotic infarct” presented intermediate mean ranks of 38.59 and 32.91, respectively.

#### 3.1.5. Treatment Modality

Regarding treatment with rt-PA, analyses revealed significant differences in initial clinical severity, measured by the NIHSS, between treated and untreated groups (U = 346, Z = −4.459, *p* < 0.001). Patients treated with rt-PA exhibited higher average scores (Mean rank = 72.96 vs. 43.05), indicating greater initial severity in this group. For the Barthel Index, both at discharge and follow-up, marginally significant differences were observed (U = 679.5, Z = −2.029, *p* = 0.042 and U = 692.5, Z = −2.001, *p* = 0.045, respectively).

Patients treated with mechanical thrombectomy showed greater initial clinical severity as measured by the NIHSS (U = 250.5, Z = −2.650, *p* = 0.008). Significant differences were observed in the Barthel Index both at discharge and follow-up (U = 314, Z = −2.383, *p* = 0.017 and U = 304, Z = −2.666, *p* = 0.008, respectively). Mean ranks were higher in the group without thrombectomy (Barthel at discharge: 51.93 vs. 34.55; follow-up: 52.05 vs. 33.64), suggesting faster functional recovery in patients not treated with thrombectomy.

Mean ranks suggest a greater emotional impact in the group treated with mechanical thrombectomy (BDI at follow-up: 68.23 vs. 47.72). This highlights a potential association between mechanical thrombectomy and heightened emotional distress during follow-up.

#### 3.1.6. Pain

In terms of emotional dimensions and pain, assessed using the BDI and pain scales, marginally significant differences were observed in BDI scores at follow-up (U = 283.5, Z = −2.240, *p* = 0.025).

### 3.2. Outcome Measures

#### 3.2.1. Neurological Function

At admission, 70% of patients exhibited cognitive impairment, as assessed by the NIHSS. Upon discharge, 54% still showed signs of cognitive deterioration, evaluated using the MEC test. During follow-up consultations, this frequency decreased to 28%, demonstrating significant improvement in cognitive performance.

**Figure 1:**
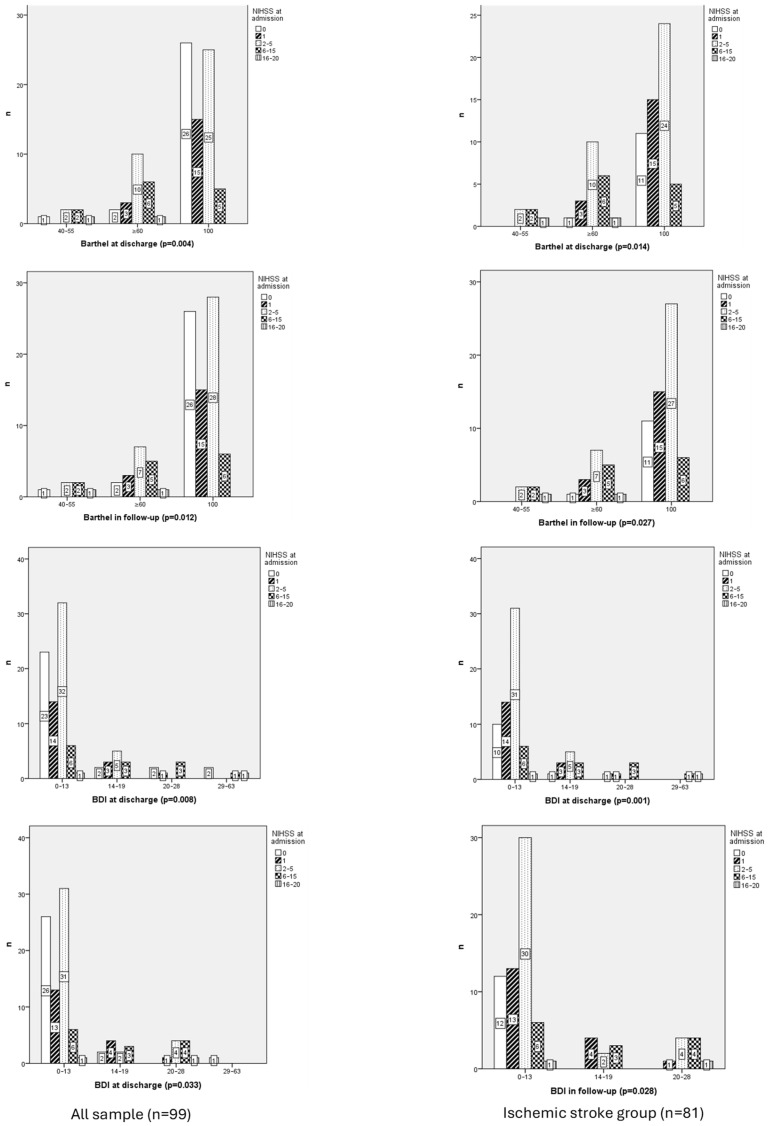
Chi-Square Correlation Table Between the Degree of Cognitive Impairment at Admission (NIHSS) and Functionality at Discharge and Follow-Up (Barthel) in whole sample vs. ischemic stroke group.

NIHSS at admission was related with Barthel at discharge (*p* = 0.004); Barthel in follow-up (0.012); BDI at discharge (*p* = 0.008); and BDI in follow-up (0.033; Figure 1).

Barthel Index scores at discharge and follow-up showed significant differences across NIHSS categories (χ^2^ = 19.165, df = 4, *p* = 0.001 and χ^2^ = 16.950, df = 4, *p* = 0.002, respectively), confirming the relationship between initial NIHSS severity and functional recovery.

Neurological status at admission, assessed via the NIHSS, demonstrated moderate negative correlations with functionality at discharge (rho = −0.361, *p* = 0.001) and functionality at follow-up (rho = −0.309, *p* = 0.002). This suggests that worse neurological status at admission is associated with lower functional capacity both at discharge and during follow-up.

BDI scores at discharge increased with NIHSS severity, ranging from 46.34 in the 0-point category to 68.58 in the 6–15-point category, though differences were not statistically significant (χ^2^ = 7.171, df = 4, *p* = 0.127). Similarly, BDI scores at follow-up showed a comparable trend, increasing from 44.31 in the 0-point category to 68.77 in the 6–15-point category, approaching statistical significance (χ^2^ = 8.368, df = 4, *p* = 0.079).

The NIHSS showed a weak positive correlation with BDI scores at follow-up (rho = 0.236, *p* = 0.019), suggesting that worse neurological status at admission may be associated with higher levels of depressive symptoms during follow-up.

**Figure 2:**
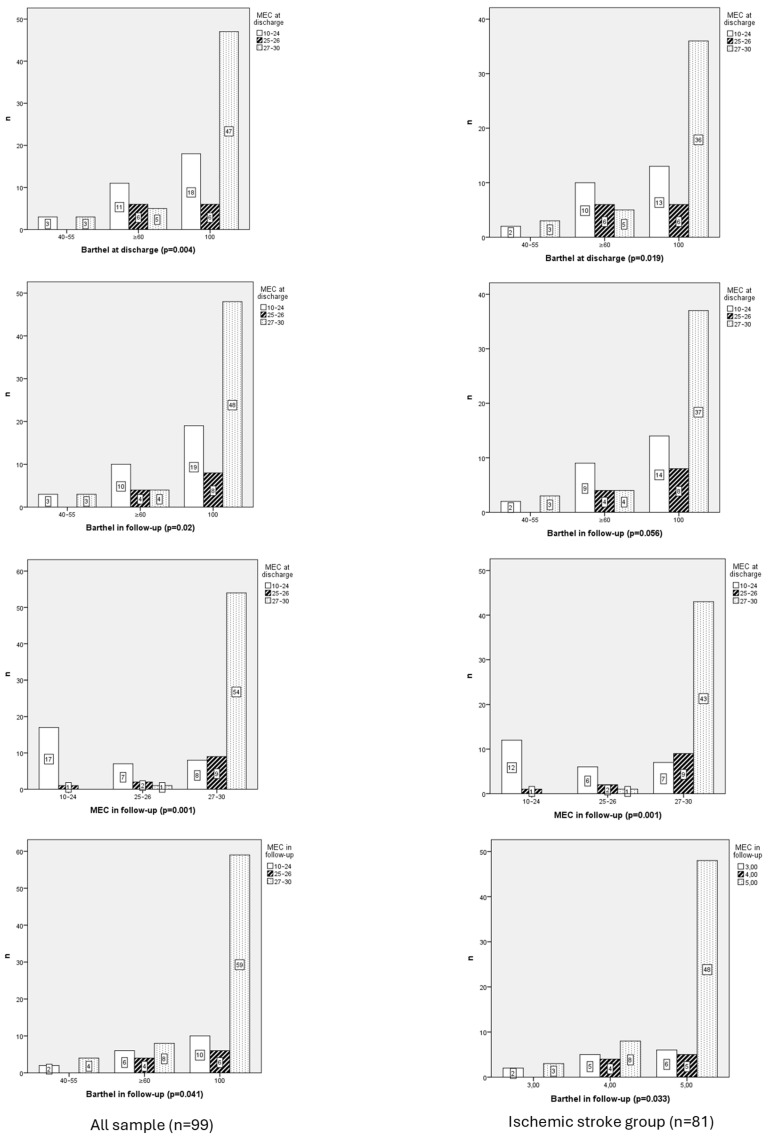
Chi-Square Correlation Table Between Cognitive Performance (MEC) at Discharge and in Follow-Up and with independence function (Barthel) in whole sample vs. ischemic stroke group.

The analysis of MEC scores at discharge and during follow-up (Figure 2) revealed significant cognitive improvement over time (*p* = 0.001). Patients with higher MEC scores at discharge (27–30) demonstrated the greatest retention of cognitive function, maintaining the same score in follow-up, whereas those with lower scores showed limited or no recovery.

When analyzing associations between MEC scores at discharge, results revealed significant relationships with MEC scores in follow-up (χ^2^ = 54.328, df = 2, *p* = 0.001).

The MEC at discharge showed a strong positive correlation with MEC in follow-up (rho = 0.778, *p* = 0.001), suggesting that worse neurological status at admission may be associated with higher levels of depressive symptoms during follow-up

MEC at discharge was related with Barthel at discharge (*p* = 0.004) and Barthel in follow-up (0.019). Also, MEC in follow-up was correlated with Barthel at discharge (0.02) and in follow-up (0.041).

MEC scores at discharge and follow-up showed statistically significant differences (χ^2^ = 10.576, df = 2, *p* = 0.005 and χ^2^ = 9.405, df = 2, *p* = 0.009, respectively), with mean ranks in follow-up increasing from 44.25 and 45.83 in the 40–55-point category to 55.10 and 54.24 in the 100-point category.

Furthermore, functionality at discharge showed moderate positive correlations with MEC scores at discharge (rho = 0.299, *p* = 0.003) and MEC scores at follow-up (rho = 0.349, *p* = 0.001), suggesting that better functionality at discharge is associated with improved cognitive performance.

Functionality at follow-up displayed similar patterns, with moderate positive correlations with Mini-Mental scores at discharge (rho = 0.290, *p* = 0.004) and MEC scores at follow-up (rho = 0.344, *p* = 0.001).

#### 3.2.2. Neurological Function and Ischemic Stroke Group

A statistically significant association was observed between the Barthel at discharge and NIHSS for ischemic stroke patients (*p* = 0.014). Additionally, a linear-by-linear association (χ2 = 14.651; *p* < 0.001) confirmed a significant linear relationship between the variables. Among patients categorized with Barthel = 3, 40% were classified under NIHSS = 2 and 40% under NIHSS 3. For Barthel = 4, 47.6% of patients fell into NIHSS 2, while 28.6% were in NIHSS = 3. In contrast, Barthel = 5 showed a predominance of NIHSS = 2 (43.6%), followed by NIHSS = 1 (27.3%). The association was further supported by negative correlations between the two variables (r = −0.413; *p* < 0.001) indicating a moderate inverse relationship.

A significant association was identified between Barthel at follow-up and NIHSS (*p* = 0.001). Additionally, a linear-by-linear association (χ^2^ = 12.112; *p* = 0.001) indicated a significant linear relationship between the variables. Within Barthel = 3, 40% of patients were classified under NIHSS = 2 and 40% under NIHSS = 3, with smaller proportions in NIHSS= 4 (20%). For Barthel = 4, most patients fell into NIHSS = 2 (41.2%), followed by NIHSS = 3 (29.4%), with smaller proportions in NIHSS = 1 (17.6%) and NIHSS = 4 (5.9%). In contrast, Barthel = 5 showed a predominance of NIHSS = 2 (45.8%) and NIHSS = 1 (25.4%), while NIHSS = 3 accounted for 10.2% and NIHSS = 4 was absent. The association was further supported by negative correlations between the two variables, with Spearman’s rank correlation (r = −0.364; *p* = 0.001), indicating a moderate inverse relationship.

A significant association was identified between BDI at discharge and NIHSS (*p* = 0.001). The linear-by-linear association (χ^2^ = 6.123; *p* = 0.013) further supported a significant linear relationship between the variables. Within BDI = 1, many patients were classified under NIHSS = 2 (50%), followed by NIHSS = 1 (22.6%) and NIHSS = 0 (16.1%), with smaller proportions in NIHSS = 3 (9.7%) and NIHSS = 4 (1.6%). For BDI = 2, NIHSS = 2 accounted for 41.7%, followed by NIHSS = 1 (25%) and NIHSS = 3 (25%). In contrast, BDI = 3 showed a predominance of NIHSS = 3 (60%), while NIHSS = 0 and NIHSS = 1 each accounted for 20%. Finally, BDI = 4 was evenly distributed between NIHSS = 3 (50%) and NIHSS = 4 (50%). The association was further supported by positive correlations between the two variables, with Spearman’s rank correlation (r = 0.223; *p* = 0.045), indicating a weak positive relationship.

A significant association was identified between BDI at discharge and NIHSS (*p* = 0.001). The linear-by-linear association (χ^2^ = 6.123; *p* = 0.013) further supported a significant linear relationship between the variables. Within BDI = 1, most patients were classified under NIHSS = 2 (50%), followed by NIHSS = 1 (22.6%) and NIHSS = 0 (16.1%), with smaller proportions in NIHSS = 3 (9.7%) and NIHSS = 4 (1.6%). For BDI = 2, NIHSS = 2 accounted for 41.7%, followed by NIHSS = 1 (25%) and NIHSS = 3 (25%). In contrast, BDI = 3 showed a predominance of NIHSS = 3 (60%), while NIHSS = 0 and NIHSS = 1 each accounted for 20%. Finally, BDI = 4 was evenly distributed between NIHSS = 3 (50%) and NIHSS = 4 (50%). The association was further supported by positive correlations between the two variables, with Spearman’s rank correlation (r = 0.223; *p* = 0.045), indicating a weak positive relationship.

A strong positive correlation was identified between Mini-Mental at discharge and Mini-Mental at follow-up (*p* < 0.001), as indicated by Spearman’s rank correlation (r = 0.686; *p* < 0.001). Patients with higher Mini-Mental scores at discharge predominantly transitioned to higher Mini-Mental categories at follow-up, reflecting consistent improvement in cognitive function.

A moderate positive correlation was identified between Barthel at follow-up and Mini-Mental at follow-up (r = 0.310; *p* = 0.005), with higher Barthel scores corresponding to better cognitive performance.

#### 3.2.3. Functional Independence

Most persons (70–75%) were fully independent in both Barthel Index assessments. The calculated Δ Barthel showed a Δ = 0.25. Among ischemic stroke patients, the Barthel Index scores at discharge were 91.11 ± 16.07 and 91.42 ± 16.17 at follow-up, reflecting a Δ = 0.31.

**Figure 3:**
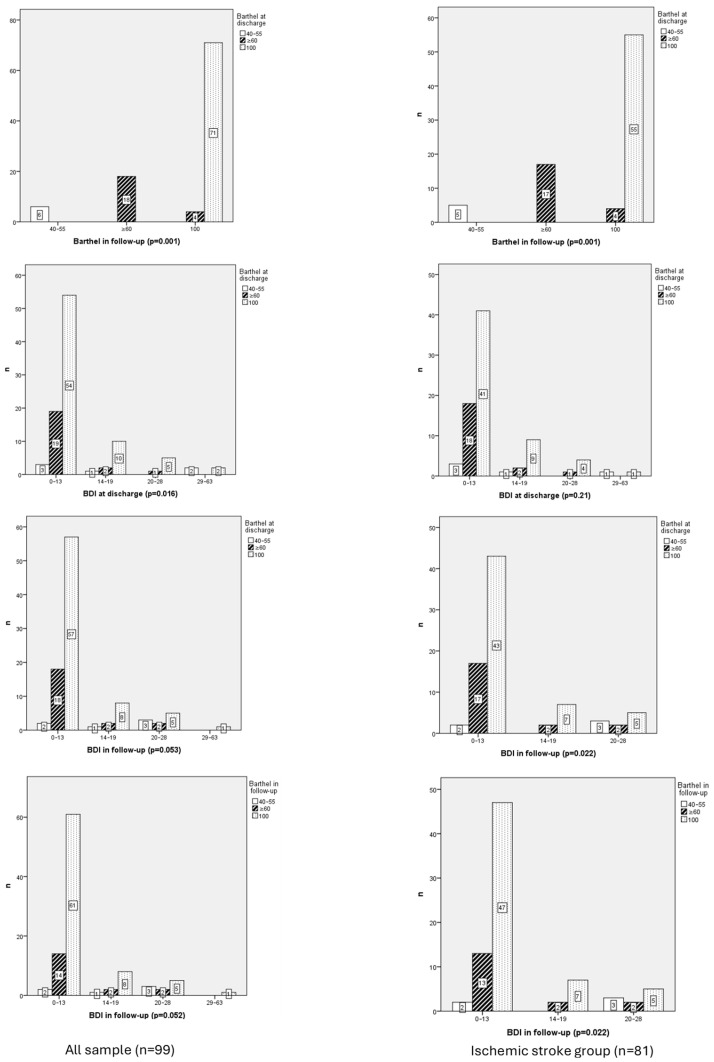
Chi-Square Correlation figures Between Functionality at Discharge and Follow-Up (Barthel) in whole sample vs. ischemic stroke group.

Barthel at discharge was related with Barthel in follow-up (0.001; Figure 3).

Barthel Index scores at discharge showed a significant increase across categories, with mean ranks rising from 3.50 in the 40–55-point category to 64.00 in the 100-point category (χ^2^ = 96.306, df = 2, *p* = 0.001). Similarly, Barthel Index scores at follow-up displayed a significant upward trend (χ^2^ = 81.775, df = 2, *p* = 0.001), with mean ranks increasing from 3.50 in the 40–55-point category to 62.00 in the 100-point category.

Functionality at discharge demonstrated a very strong positive correlation with functionality at follow-up (rho = 0.944, *p* = 0.001), indicating that patients with better functional recovery at discharge tend to maintain high levels of functionality during follow-up.

Barthel at discharge was related with BDI at discharge (*p* = 0.016) but not in follow-up (*p* = 0.053).

Emotional variables yielded mixed results. BDI scores at discharge decreased from a mean rank of 65.58 in the 40–55-point category to 50.23 in the 100-point category, although differences were not statistically significant.

#### 3.2.4. Functional Independence and Ischemic Stroke Group

A strong positive correlation was identified between functional independence at discharge and at follow-up (r = 0.904; *p* < 0.001). Significant differences between groups were analyzed (χ^2^ = 142.126; df = 4; *p* < 0.001); higher Barthel scores consistently achieved greater functional independence at discharge, underscoring the predictive value of initial functional status.

A weak negative correlation was detected between Barthel and BDI at follow-up (r = −0.253; *p* = 0.022). Differences between groups were found (χ^2^ = 11.458; df = 4; *p* = 0.022); lower depression scores tended to exhibit higher levels of functional independence. No differences were found between Barthel and BDI at discharge.

#### 3.2.5. Emotional Function

Regarding depressive symptoms, 77% of patients scored below 13 points on the BDI scale during the initial assessment, indicating minimal depressive symptoms. At follow-up, 78% of patients maintained scores below 13 points.

The analysis of the 21 items of the BDI shows that most patients were classified under the minimal depression category both at hospital discharge and during the follow-up (Table 2). However, some items, such as loss of libido, mood, and changes in sleep patterns, showed greater variability across mild, moderate, and severe depression categories. Overall, symptoms such as concentrating difficulty and fatigue or tiredness remained predominantly in the minimal category, while others displayed a broader distribution across higher categories.

**Figure 4:**
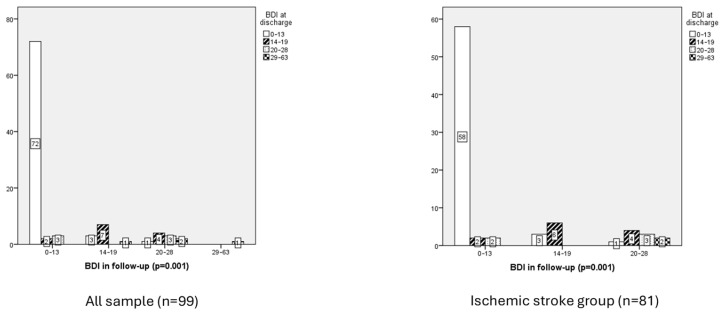
Chi-Square Correlation figure Between the Risk of Depression Symptoms (BDI) at Discharge and Follow-Up in whole sample vs. ischemic stroke group.

Depressive symptoms at discharge showed a significant relation with depressive symptoms at follow-up (*p* = 0.001), indicating that subjects with higher levels of depression at discharge tend to maintain elevated levels of depressive symptoms during follow-up (Figure 4).

The analysis of depressive symptom scores at discharge and follow-up revealed significant differences across groups, indicating a strong association between initial depressive status and subsequent emotional outcomes. The results demonstrated a statistically significant chi-square value (χ^2^ = 58.771, df = 3, *p* = 0.001), confirming variability in follow-up BDI categories based on discharge BDI scores. Patients classified in the minimal depression category at discharge (*n* = 76) had the lowest mean rank (41.45), whereas those with severe depression at discharge (*n* = 4) exhibited the highest mean rank (92.25). Intermediate categories (14–19 and 20–28 points) displayed mean ranks of 79.46 and 66.25, respectively.

Depressive symptoms at discharge showed a very strong positive correlation with depressive symptoms at follow-up (rho = 0.791, *p* = 0.001), indicating that population with higher levels of depression at discharge tend to maintain elevated levels of depressive symptoms during follow-up.

Emotional function and ischemic stroke group

The distribution of depressive symptoms revealed that most participants fell into category 1 (76.5%), indicating minimal depression, while fewer individuals were classified in categories 2, 3, and 4, corresponding to mild, moderate, and severe depression, respectively. However, the mean ranks suggest a trend toward higher depressive symptoms in the severe depression group, particularly during follow-up (mean rank: 46.68), compared to lower ranks observed in categories 1, 2, and 3.

## 4. Discussion

This study uniquely contributes to the literature by including patients with TIA alongside those with ischemic stroke, allowing for a broader understanding of recovery patterns. Additionally, the integration of cognitive, functional, and emotional assessments provides a comprehensive view of patient outcomes, highlighting the importance of multidimensional approaches in stroke care [23].

The sample in this study included 99 subjects, of whom 81.8% presented with ischemic stroke. Although functional differences, assessed using the Barthel Index, tend to diminish over time, significantly lower functional and cognitive recovery was observed in older patients, reflected in lower scores on both the Barthel Index and the MEC [24,25].

Ischemic stroke subtypes were categorized as follows: atherothrombotic infarct (12.1%), cardioembolic stroke (24.2%), essential cerebral infarct (44.4%), infarct of unusual etiology (7.1%), and lacunar infarct (12.1%). These findings suggest that cognitive outcomes, as assessed by the Mini-Mental State Examination during follow-up, may vary across ischemic stroke subtypes, warranting further investigation to better understand the underlying mechanisms contributing to these differences. This classification highlights the heterogeneity within ischemic stroke populations and underscores the need for tailored approaches to address the unique pathophysiological and clinical features of each subtype [4].

An essential line of future research would be to evaluate the impact of differences between lacunar and non-lacunar ischemic strokes. Evidence suggests that lacunar ischemic strokes, which often result from small vessel disease, differ significantly from other ischemic stroke subtypes in terms of pathophysiology, prognosis, and clinical features, emphasizing the need for translational research and advanced neuroimaging techniques to better characterize cerebral small vessel disease markers and include them in large multicenter studies. This approach could lead to improved diagnostic accuracy and more effective interventions tailored to lacunar stroke populations [4,26].

The average age was 72.43 ± 11.72 years, and many participants were men (65.7%). When comparing these demographic characteristics with previous research, relevant differences were identified. Recent studies emphasize that demographic factors such as age and sex significantly influence recovery trajectories, with older people and men often exhibiting slower functional recovery due to higher prevalence of comorbidities and vascular risk factors [27].

The study by Pego Pérez et al. [28] analyzed a larger sample of 674 subjects, with a higher mean age (73.99 ± 11.69 years) and a lower percentage of men (57%). In contrast, Sánchez Silverio et al. [29] reported a mean age of 56.6 ± 12.6 years, but with a gender distribution of 46.8% women vs. 53.2% men (*n* = 62). These comparisons highlight the variability in demographic characteristics across studies, underscoring the importance of the population context in interpreting results.

Additionally, global differences between men and women were identified, although no individual variables, such as NIHSS or Barthel, showed statistical significance. This may reflect underlying biological or social factors. Patients undergoing mechanical thrombectomy exhibited a higher risk of developing depressive symptoms, which may be partially attributed to the psychological impact of being transferred to specialized centers in Coruña or Santiago de Compostela. The necessity of relocation to another province could exacerbate emotional distress, as it disrupts social support systems and heightens feelings of vulnerability during the acute phase of care.

Cognitive status in this study was assessed three different times. Initially, upon hospital admission, the mean score was 2.72 ± 3.56 points, indicating mild cognitive impairment. Comparing these findings with other studies reveals notable variability in results. Recent guidelines emphasize the importance of combining neuroimaging techniques with standardized cognitive assessments to improve early detection and intervention strategies for cognitive impairment post-stroke [30]. Bermello López et al. [7] reported a mean score of 12.1 ± 7.3 points, reflecting moderate impairment, while Pego Pérez et al. [31] recorded a mean of 16.2 ± 7.4 points, corresponding to significant impairment. In subsequent evaluations conducted at discharge and during follow-up consultations using the MEC, the mean scores were 25.38 ± 4.57 and 27.03 ± 4.02 points, respectively, indicating a questionable cognitive status or absence of impairment. Similar results were observed in the study by Sánchez Silverio et al. [29], with a mean of 24.9 ± 4.1 points, also reflecting a questionable cognitive status. These findings emphasize the diversity in results depending on the context and the tools used for cognitive assessment.

This study identified a correlation between patient cognitive status at admission and their subsequent functionality, observing that greater cognitive impairment at admission is associated with poorer functional prognosis. Similar results were reported by Bermello López et al. [7], who concluded that the NIHSS is a good predictor of functionality three months after stroke. These findings suggest that cognitive impairment may hinder recovery by limiting patient ability to engage in rehabilitation programs or adapt to functional challenges. Early cognitive screening and targeted interventions could enhance rehabilitation outcomes and mitigate long-term disability.

Additionally, a relationship was found between initial cognitive status at discharge and the emergence of subsequent depressive symptoms. Specifically, greater cognitive impairment at admission was associated with more pronounced depressive symptoms in later stages. Carnés-Vendrell et al. [32] also addressed this connection, noting that the relationship between post-stroke cognitive impairment and post-stroke depression is controversial and appears to be bidirectional: greater cognitive impairment increases the risk of depression, but likewise, greater depression heightens the risk of cognitive impairment. These findings underscore the importance of integrated approaches that address both cognitive and emotional health in stroke patients, as targeting one domain may indirectly benefit the other.

Voxel-Based Morphometry (VBM) has proven to be a valuable tool in quantifying brain changes, particularly in gray and white matter, that contribute to cognitive impairment in patients with acute small vessel disease. VBM can identify specific regions of gray-matter shrinkage, such as the hippocampus, parahippocampal gyri, and temporal lobes, which correlate with cognitive deficits in vascular-related mild cognitive impairment. Furthermore, VBM facilitates the analysis of structural brain changes beyond subcortical damage, offering insights into the broader impact of cerebrovascular pathology on cognitive function. This technique reinforces the importance of integrating advanced neuroimaging methods in stroke research to enhance early detection and improve intervention strategies for cognitive impairment [26].

The variability in results across studies may be influenced by differences in sample characteristics, such as age distribution, stroke severity, and inclusion criteria. Additionally, cultural and healthcare system differences could play a role in shaping recovery trajectories and patient outcomes. Standardizing methodologies and expanding cross-cultural research are essential to address these discrepancies [27].

In terms of functionality, a mean score of 91.92 ± 15.60 was obtained at discharge and 92.17 ± 15.67 during follow-up (independence/mild dependence). The delta of 0.25 reflected a slight but significant improvement in the functionality of stroke patients, particularly those with low scores at discharge, enabling them to perform basic activities that impact their independence and quality of life. This change, consistent with previous studies, highlights the importance of early and continuous interventions that integrate physical, emotional, and cognitive aspects to maximize recovery. Furthermore, it underscores the need to establish realistic goals, as even modest progress can be crucial for patient adaptation and autonomy.

When compared with other studies, some differences in dependency levels were identified. In the study by Pego Pérez et al. [31], the mean score was 78.84 ± 24.42 points, indicating a slightly higher level of dependency than observed in this study (mild dependency). Similarly, Sánchez Silverio et al. [29] reported a mean score of 78.1 ± 14 points, also corresponding to mild dependency. These discrepancies could be explained by differences in sample composition, as previous studies included only patients with ischemic stroke, whereas this study also included individuals who suffered TIA.

Regarding depressive symptoms, the results of this study showed a mean score of 9.05 ± 8.56 points at discharge and 8.27 ± 7.56 points during the follow-up consultation, both corresponding to a minimal level of depression. Similarly, the study by Geun-Young Park et al. [11] reported a mean score of 12.9 ± 11.1 points. Also, the BDI scores were low, which could be explained by the early timing of the evaluations, conducted during the acute or subacute phase. This finding aligns with the literature, which indicates that post-stroke depressive symptoms tend to develop or become more apparent several months after the initial event [11].

The results show the prevalence of minimal depression in the ischemic stroke population while underscoring the need for targeted interventions for individuals presenting severe depressive symptoms. The results reflect a general trend toward improvement in depressive symptoms after hospital discharge, with most patients classified under the minimal depression category. However, the persistence of symptoms such as loss of libido, mood, and changes in sleep patterns in higher categories highlight areas that may require specific interventions. Additionally, the increase in mild and moderate depression categories for certain items during the follow-up consultation suggests that some emotional and physical aspects were not fully resolved with initial treatment. These findings emphasize the need for personalized therapeutic strategies and closer monitoring to ensure comprehensive recovery.

The findings underscore the need for a multidimensional approach in the evaluation and follow-up of patients with stroke. Current evidence-based guidelines advocate multidisciplinary teams to address neurological, functional, and emotional dimensions simultaneously, ensuring comprehensive care and minimizing disparities in treatment outcomes [23].

The Registered Nurses’ Association of Ontario stands out for its leadership in creating guidelines aimed at fostering a comprehensive approach to patient care, with the goal of minimizing disparities in treatment and optimizing health outcomes. These guidelines, developed, reviewed, and periodically updated, serve as essential resources for standardizing and improving patient care after a stroke. Additionally, the adoption of these care standards would significantly contribute to the more efficient management of available healthcare resources [13,16].

It is crucial to advance toward the standardization of care through the progressive integration of recommendations based on nursing best practice guidelines. This would enable the evaluation of the impact of nursing care and interventions on stroke patient recovery, adopting a holistic approach that prioritizes health education. Moreover, expanding data collection and extending follow-up periods is necessary, as most current studies focus predominantly on acute and subacute phases, often overlooking the stabilization stage of the disease.

Future research should focus on implementing specific interventions such as the use of robotic-assisted rehabilitation (e.g., Lokomat for gait training) and cognitive training programs like the Cognitive Orientation to Daily Occupational Performance (CO-OP) approach, which have shown promising results in improving functional and cognitive recovery in stroke survivors. Additionally, studies exploring the long-term impact of pharmacological therapies, such as selective serotonin reuptake inhibitors (SSRIs) for post-stroke depression, could provide valuable insights into optimizing recovery strategies during the chronic phase of the disease. Multi-center trials evaluating these approaches in diverse populations would be vital to establish standardized practices and reduce disparities in care.

## 5. Limitations

This study presented several limitations that must be considered when interpreting the results. Firstly, it involved a specific cohort, which may restrict the generalizability of certain findings. Additionally, the lack of long-term follow-up prevented the evaluation of the evolution of the analyzed variables beyond the subacute phase, which could have provided valuable insights into functional and emotional stabilization in later stages.

Although various clinical and emotional variables were included, other potentially relevant factors, such as social support, comorbidities, and access to rehabilitation resources, were not considered, and these may have influenced the outcomes. Finally, the observational design of the study did not allow for definitive causal relationships to be established between the variables analyzed.

## 6. Conclusions

Greater cognitive impairment at hospital admission was associated with poorer functional prognosis. Additionally, greater cognitive impairment at admission correlated with more pronounced depressive symptoms in later stages. Loss of functionality at discharge was linked to higher levels of depression during follow-up. Older people demonstrated significantly lower functional and cognitive recovery compared to younger people.

This study provides strong evidence for the integration of multidimensional assessments into stroke care. Future efforts should focus on extending follow-up periods and personalizing interventions to address the unique cognitive, functional, and emotional needs of each patient.

## Figures and Tables

**Table 1:** Frequency Table and Measures of Central Tendency and Dispersion for the Characteristics of the Variables Studied.

	*n*	%	Mean	SD	P25	P50	P75	IL	SL
Age			72.43	11.72	65	64	82	41	95
<65	23	23.2							
65–80	46	46.5							
>80	30	30.3							
Sex									
Man	65	65.7							
Women	34	34.3							
Type of stroke									
Ischemic	81	81.8							
TIA	18	18.2							
Ischemic stroke subtypes									
Atherothrombotic infarct	12	12.1							
Cardioembolic stroke	24	24.2							
Essential cerebral infarct	44	44.4							
Infarct of unusual etiology	7	7.1							
Lacunar infarct	12	12.1							
rt-PA									
No	76	76.8							
Yes	23	23.2							
Thrombectomy									
No	88	88.9							
Yes	11	11.1							
PU									
No	99	100							
Yes	0	0							
Dysphagia Test									
No	0	0							
Yes	99	100							
Dysphagia									
No	98	99							
Yes	1	1							
Aspiration Pneumonia									
No	98	99							
Yes	1	1							
Pain			0.36	1.26				0	7
0	89	89.9							
1	1	1.0							
2	3	3.0							
3	1	1.0							
4	1	1.0							
5	3	3.0							
6	0	0							
7	1	1.0							
8	0	0							
9	0	0							
10	0	0							
NIHSS			2.72	3.56	0	2	3	0	20
0	29	29.3							
1	18	18.2							
2–5	37	37.4							
6–15	13	13.1							
16–20	2	2.0							
>20	0	0							
Basal Barthel			98.5	7.2	100	100	100	55	100
100	93	93.9							
≥60	4	4.1							
40–55	2	2							
20–35	0	0							
<20	0	0							
Barthel at discharge			91.92	15.60	95	100	100	40	100
100	71	71.7							
≥60	22	22.2							
40–55	6	6.1							
20–35	0	0							
<20	0	0							
Barthel Index in Follow-Up			92.17	15.67	100	100	100	40	100
100	75	75.8							
≥60	18	18.2							
40–55	6	6.1							
20–35	0	0							
<20	0	0							
BDI at discharge			9.05	8.56	3	7	13	0	43
0–13	76	76.8							
14–19	13	13.1							
20–28	6	6.1							
29–63	4	4.0							
BDI in follow-up			8.27	7.56	1	7	13	0	34
0–13	77	77.8							
14–19	11	11.1							
20–28	10	10.1							
29–63	1	1.0							
Mini-Mental at discharge			25.38	4.57	23	27	29	10	30
27–30	55	55.6							
25–26	12	12.1							
10–24	32	32.3							
6–9	0	0							
<6	0	0							
Mini-Mental in follow-up			27.03	4.02	26	28	30	12	30
27–30	71	71.7							
25–26	10	10.1							
10–24	18	18.2							
6–9	0	0							
<6	0	0							
Falls									
No	97								
Yes	2								
Temperature			36.29	0.44	36	36.3	36.6	35	37.3
Systolic blood pressure			146.25	25.15	129	148	162	96	218
Diastolic blood pressure			78.01	12.56	70	78	85	48	114
Mean arterial pressure (MAP)			112.13	16.48	101	111	123.5	73	154
Heart rate			69.59	12.8	60	68	78	45	110
Glucose			112.61	38.71	93	103	120	72	349
Stay in hospital (days)			7.1	5.2	3	6	9	1	32
0–3	25	25.3							
4–6	30	30.3							
7–8	17	17.2							
≥9	27	27.3							

*n*: sample (*n* = 99); %: frequency; SD: standard deviation; P25. P50. P75: percentile; IL: inferior limit; SL: superior limit.

**Table 2:** (A) Distribution by Categories of Symptoms from the Beck Depression Inventory at Discharge. (B) Distribution by Categories of Symptoms from the Beck Depression Inventory in follow-up.

(A)							
Item number	Symptom	Severity		BDI at discharge, *n* (%)			
				Minimal	Mild	Moderate	Severe
1	Pessimism		Minimal	56 (86.2)	6 (9.2)	2 (3.1)	1 (1.5)
			Mild	18 (66.7)	6 (22.2)	2 (7.4)	1 (3.7)
			Moderate	0 (0)	1 (33.3)	1 (33.3)	1 (33.3)
			Severe	2 (50)	0 (0)	1 (25)	1 (25)
2	Mood		Minimal	63 (88.7)	5 (7)	2 (2.8)	1 (1.4)
			Mild	10 (41.7)	8 (33.3)	4 (16.7)	2 (8.3)
			Moderate	2 (100)	0 (0)	0 (0)	0 (0)
			Severe	1 (50)	0 (0)	0 (0)	1 (50)
3	Failure		Minimal	71 (82.6)	11 (12.8)	3 (3.5)	1 (1.2)
			Mild	5 (55.6)	1 (11.1)	2 (22.2)	1 (11.1)
			Moderate	0 (0)	1 (33.3)	1 (33.3)	1 (33.3)
			Severe	0 (0)	0 (0)	0 (0)	1 (100)
4	Dissatisfaction		Minimal	57 (87.7)	7 (10.8)	1 (1.5)	0 (0)
			Mild	17 (54.8)	6 (19.4)	5 (16.1)	3 (9.7)
			Moderate	2 (66.7)	0 (0)	0 (0)	1 (33.3)
			Severe	0 (0)	0 (0)	0 (0)	0 (0)
5	Feelings of guilt		Minimal	66 (85.7)	9 (11.7)	2 (2.6)	0 (0)
			Mild	9 (45)	4 (20)	4 (20)	3 (15)
			Moderate	1 (50)	0 (0)	0 (0)	1 (50)
			Severe	0 (0)	0 (0)	0 (0)	0 (0)
6	Feelings of punishment		Minimal	71 (84.5)	10 (11.9)	3 (3.6)	0 (0)
			Mild	5 (55.6)	1 (11.1)	2 (22.2)	1 (11.1)
			Moderate	0 (0)	1 (100)	0 (0)	0 (0)
			Severe	0 (0)	1 (20)	1 (20)	3 (60)
7	Self-disapproval		Minimal	73 (84.9)	8 (9.3)	5 (5.8)	0 (0)
			Mild	3 (42.9)	3 (42.9)	1 (14.3)	0 (0)
			Moderate	0 (0)	1 (20)	0 (0)	4 (80)
			Severe	0 (0)	1 (100)	0 (0)	0 (0)
8	Self-criticism		Minimal	68 (89.5)	6 (7.9)	2 (2.6)	0 (0)
			Mild	7 (50)	5 (35.7)	2 (14.3)	0 (0)
			Moderate	1 (12.5)	2 (25)	2 (25)	3 (37.5)
			Severe	0 (0)	0 (0)	0 (0)	1 (100)
9	Suicidal thoughts or ideas		Minimal	73 (81.1)	9 (10)	5 (5.6)	3 (3.3)
			Mild	3 (33.3)	4 (44.4)	1 (11.1)	1 (11.1)
			Moderate	0 (0)	0 (0)	0 (0)	0 (0)
			Severe	0 (0)	0 (0)	0 (0)	0 (0)
10	Crying		Minimal	66 (84.6)	9 (11.5)	2 (2.6)	1 (1.3)
			Mild	7 (50)	4 (28.6)	3 (21.4)	0 (0)
			Moderate	3 (75)	0 (0)	1 (25)	0 (0)
			Severe	0 (0)	0 (0)	0 (0)	3 (100)
11	Fatigability		Minimal	58 (92.1)	5 (7.9)	0 (0)	0 (0)
			Mild	13 (52)	7 (28)	5 (20)	0 (0)
			Moderate	2 (50)	1 (25)	0 (0)	1 (25)
			Severe	3 (42.9)	0 (0)	1 (14.3)	3 (42.9)
12	Social withdrawal		Minimal	65 (82.3)	10 (12.7)	2 (2.5)	2 (2.5)
			Mild	11 (64.7)	2 (11.8)	3 (17.6)	1 (5.9)
			Moderate	0 (0)	0 (0)	0 (0)	0 (0)
			Severe	0 (0)	1 (33.3)	1 (33.3)	1 (33.3)
13	Indecisiveness		Minimal	65 (91.5)	6 (8.5)	0 (0)	0 (0)
			Mild	10 (50)	6 (30)	3 (15)	1 (5)
			Moderate	1 (16.7)	1 (16.7)	2 (33.3)	2 (33.3)
			Severe	0 (0)	0 (0)	1 (50)	1 (50)
14	Changes in physical appearance		Minimal	70 (90.9)	5 (6.5)	2 (2.6)	0 (0)
			Mild	6 (35.3)	7 (41.2)	3 (17.6)	1 (5.9)
			Moderate	0 (0)	0 (0)	1 (33.3)	2 (66.7)
			Severe	0 (0)	1 (50)	0 (0)	1 (50)
15	Loss of energy		Minimal	30 (96.8)	1 (3.2)	0 (0)	0 (0)
			Mild	41 (80.4)	8 (15.7)	2 (3.9)	0 (0)
			Moderate	5 (33.3)	4 (26.7)	4 (26.7)	2 (13.3)
			Severe	0 (0)	0 (0)	0 (0)	2 (100)
16	Changes in sleep patterns		Minimal	41 (91.1)	4 (8.9)	0 (0)	0 (0)
			Mild	27 (79.4)	4 (11.8)	1 (2.9)	2 (5.9)
			Moderate	8 (57.1)	2 (14.3)	3 (21.4)	1 (7.1)
			Severe	0 (0)	3 (50)	2 (33.3)	1 (16.7)
17	Irritability		Minimal	64 (88.9)	6 (8.3)	2 (2.8)	0 (0)
			Mild	11 (52.4)	5 (23.8)	2 (9.5)	3 (14.3)
			Moderate	0 (0)	0 (0)	2 (100)	0 (0)
			Severe	1 (25)	2 (50)	0 (0)	1 (25)
18	Loss of appetite		Minimal	62 (88.6)	4 (5.7)	2 (4.3)	1 (1.4)
			Mild	13 (56.5)	6 (26.1)	2 (8.7)	2 (8.7)
			Moderate	1 (20)	2 (40)	1 (20)	1 (20)
			Severe	0 (0)	1 (100)	0 (0)	0 (0)
19	Difficulty concentrating		Minimal	52 (100)	0 (0)	0 (0)	0 (0)
			Mild	22 (61.1)	10 (27.8)	4 (11.1)	0 (0)
			Moderate	2 (22.2)	3 (33.3)	2 (22.2)	2 (22.2)
			Severe	0 (0)	0 (0)	0 (0)	2 (100)
20	Fatigue or tiredness		Minimal	36 (97.3)	1 (2.7)	0 (0)	0 (0)
			Mild	31 (70.5)	10 (22.7)	2 (4.5)	1 (2.3)
			Moderate	8 (61.5)	2 (15.4)	2 (15.4)	1 (7.7)
			Severe	1 (20)	0 (0)	2 (40)	2 (40)
21	Loss of libido		Minimal	50 (94.3)	2 (3.8)	1 (1.9)	0 (0)
			Mild	17 (81)	4 (19)	0 (0)	0 (0)
			Moderate	4 (44.4)	1 (11.1)	2 (22.2)	2 (22.2)
			Severe	5 (31.3)	6 (37.5)	3 (18.8)	2 (12.5)
(B)				BDI in follow-up, *n* (%)			
				Minimal	Mild	Moderate	Severe
1	Pessimism		Minimal	61 (89.7)	4 (5.9)	2 (2.9)	1 (1.5)
			Mild	16 (57.1)	7 (25)	5 (17.9)	0 (0)
			Moderate	0 (0)	0 (0)	2 (100)	0 (0)
			Severe	0 (0)	0 (0)	1 (100)	0 (0)
2	Mood		Minimal	68 (90.7)	4 (5.3)	2 (7.7)	1 (1.3)
			Mild	7 (33.3)	6 (28.6)	8 (38.1)	0 (0)
			Moderate	1 (50)	1 (50)	0 (0)	0 (0)
			Severe	1 (100)	0 (0)	0 (0)	0 (0)
3	Failure		Minimal	74 (85.1)	8 (9.2)	4 (4.6)	1 (1.1)
			Mild	3 (30)	3 (30)	4 (40)	0 (0)
			Moderate	0 (0)	0 (0)	1 (100)	0 (0)
			Severe	0 (0)	0 (0)	1 (100)	0 (0)
4	Dissatisfaction		Minimal	60 (92.3)	2 (3.1)	3 (4.6)	0 (0)
			Mild	16 (51.6)	9 (29)	5 (16.1)	1 (3.2)
			Moderate	1 (33.3)	0 (0)	2 (66.7)	0 (0)
			Severe	0 (0)	0 (0)	0 (0)	0 (0)
5	Feelings of guilt		Minimal	70 (84.3)	6 (7.2)	7 (8,4)	0 (0)
			Mild	7 (46.7)	5 (33.3)	3 (20)	0 (0)
			Moderate	0 (0)	0 (0)	0 (0)	1 (100)
			Severe	0 (0)	0 (0)	0 (0)	0 (0)
6	Feelings of punishment		Minimal	75 (83.3)	8 (8.9)	7 (7.8)	0 (0)
			Mild	2 (40)	2 (40)	1 (20)	0 (0)
			Moderate	0 (0)	1 (50)	0 (0)	1 (50)
			Severe	0 (0)	0 (0)	2 (100)	0 (0)
7	Self-disapproval		Minimal	72 (83.7)	8 (9.3)	6 (7)	0 (0)
			Mild	4 (44.4)	3 (33.3)	2 (22.2)	0 (0)
			Moderate	1 (25)	0 (0)	2 (50)	1 (25)
			Severe	0 (0)	0 (0)	0 (0)	0 (0)
8	Self-criticism		Minimal	71 (85.5)	7 (8.4)	5 (6)	0 (0)
			Mild	6 (54.5)	3 (27.3)	2 (18.2)	0 (0)
			Moderate	0 (0)	1 (25)	3 (75)	0 (0)
			Severe	0 (0)	0 (0)	0 (0)	1 (100)
9	Suicidal thoughts or ideas		Minimal	74 (80.4)	9 (9.8)	8 (8.7)	1 (1.1)
			Mild	3 (42.9)	2 (28.6)	2 (28.6)	0 (0)
			Moderate	0 (0)	0 (0)	0 (0)	0 (0)
			Severe	0 (0)	0 (0)	0 (0)	0 (0)
10	Crying		Minimal	64 (94.1)	3 (4.4)	1 (1.5)	0 (0)
			Mild	11 (39.3)	8 (28.6)	9 (32.1)	0 (0)
			Moderate	2 (100)	0 (0)	0 (0)	0 (0)
			Severe	0 (0)	0 (0)	0 (0)	1 (100)
11	Fatigability		Minimal	64 (94.1)	3 (4.4)	1 (1.5)	0 (0)
			Mild	11 (39.3)	8 (28.6)	9 (32.1)	0 (0)
			Moderate	2 (100)	0 (0)	0 (0)	0 (0)
			Severe	0 (0)	0 (0)	0 (0)	1 (100)
12	Social withdrawal		Minimal	64 (90.1)	4 (5.6)	2 (2.8)	1 (1.4)
			Mild	13 (54.2)	6 (25)	5 (20.8)	0 (0)
			Moderate	0 (0)	1 (33.3)	2 (66.7)	0 (0)
			Severe	0 (0)	0 (0)	1 (100)	0 (0)
13	Indecisiveness		Minimal	65 (94.2)	4 (5.8)	0 (0)	0 (0)
			Mild	12 (50)	6 (25)	5 (20.8)	1 (4.2)
			Moderate	0 (0)	1 (20)	4 (80)	0 (0)
			Severe	0 (0)	0 (0)	1 (100)	0 (0)
14	Changes in physical appearance		Minimal	65 (90.3)	3 (4.2)	4 (5.6)	0 (0)
			Mild	10 (47.6)	7 (33.3)	4 (19)	0 (0)
			Moderate	2 (40)	1 (20)	2 (40)	0 (0)
			Severe	0 (0)	0 (0)	0 (0)	1 (100)
15	Loss of energy		Minimal	35 (100)	0 (0)	0 (0)	0 (0)
			Mild	39 (81.3)	7 (14.6)	2 (4.2)	0 (0)
			Moderate	3 (23.1)	4 (30.8)	6 (46.2)	0 (0)
			Severe	0 (0)	0 (0)	2 (66.7)	1 (33.3)
16	Changes in sleep patterns		Minimal	48 (90.6)	3 (5.7)	2 (3.8)	0 (0)
			Mild	21 (77.8)	4 (14.8)	2 (7.4)	0 (0)
			Moderate	7 (46.7)	3 (20)	5 (33.3)	0 (0)
			Severe	1 (25)	1 (25)	1 (25)	1 (25)
17	Irritability		Minimal	58 (89.2)	3 (4.6)	4 (6.2)	0 (0)
			Mild	18 (58.1)	8 (25.8)	5 (16.1)	0 (0)
			Moderate	1 (100)	0 (0)	0 (0)	0 (0)
			Severe	0 (0)	0 (0)	1 (50)	1 (50)
18	Loss of appetite		Minimal	63 (88.7)	4 (5.6)	4 (5.6)	0 (0)
			Mild	14 (63.6)	4 (18.2)	3 (13.6)	1 (4.5)
			Moderate	0 (0)	3 (50)	3 (50)	0 (0)
			Severe	0 (0)	0 (0)	0 (0)	0 (0)
19	Difficulty concentrating		Minimal	49 (94.2)	1 (1.9)	2 (3.8)	0 (0)
			Mild	26 (68.4)	8 (21.1)	4 (10.5)	0 (0)
			Moderate	2 (22.2)	2 (22.2)	4 (44.4)	1 (11.1)
			Severe	0 (0)	0 (0)	0 (0)	0 (0)
20	Fatigue or tiredness		Minimal	36 (97.3)	0 (0)	1 (2.7)	0 (0)
			Mild	37 (80.4)	8 (17.4)	1 (2.2)	0 (0)
			Moderate	3 (25)	3 (25)	6 (50)	0 (0)
			Severe	1 (25)	0 (0)	2 (52)	1 (50)
21	Loss of libido		Minimal	49 (90.7)	2 (3.7)	3 (5.6)	0 (0)
			Mild	15 (75)	5 (25)	0 (0)	0 (0)
			Moderate	3 (42.9)	2 (28.6)	1 (14.3)	1 (14.3)
			Severe	10 (55.6)	2 (11.1)	6 (33.3)	0 (0)

*n*: 99; %: frequency.

## Data Availability

The datasets generated and/or analyzed during the current study are not publicly available but can be obtained from the corresponding author upon reasonable request, subject to privacy and ethical restrictions.
